# The application of carbon nanotubes in target drug delivery systems for cancer therapies

**DOI:** 10.1186/1556-276X-6-555

**Published:** 2011-10-13

**Authors:** Wuxu Zhang, Zhenzhong Zhang, Yingge Zhang

**Affiliations:** 1 Institute of Pharmacology and Toxicology and Key Laboratory of Nanopharmacology and Nanotoxicology, Beijing Academy of Medical Science, Zhengzhou, Henan, People's Republic of China; 2 Nanotechnology Research Center for Drugs, Zhengzhou University, Zhengzhou, Henan, People's Republic of China

**Keywords:** carbon nanotubes, cancer therapies, drug delivery systems, target chemotherapy

## Abstract

Among all cancer treatment options, chemotherapy continues to play a major role in killing free cancer cells and removing undetectable tumor micro-focuses. Although chemotherapies are successful in some cases, systemic toxicity may develop at the same time due to lack of selectivity of the drugs for cancer tissues and cells, which often leads to the failure of chemotherapies. Obviously, the therapeutic effects will be revolutionarily improved if human can deliver the anticancer drugs with high selectivity to cancer cells or cancer tissues. This selective delivery of the drugs has been called target treatment. To realize target treatment, the first step of the strategies is to build up effective target drug delivery systems. Generally speaking, such a system is often made up of the carriers and drugs, of which the carriers play the roles of target delivery. An ideal carrier for target drug delivery systems should have three pre-requisites for their functions: (1) they themselves have target effects; (2) they have sufficiently strong adsorptive effects for anticancer drugs to ensure they can transport the drugs to the effect-relevant sites; and (3) they can release the drugs from them in the effect-relevant sites, and only in this way can the treatment effects develop. The transporting capabilities of carbon nanotubes combined with appropriate surface modifications and their unique physicochemical properties show great promise to meet the three pre-requisites. Here, we review the progress in the study on the application of carbon nanotubes as target carriers in drug delivery systems for cancer therapies.

## Introduction

Cancers are a kind of the diseases that are hardest to cure, and most cancer patients definitely die even when treated with highly developed modern medicinal techniques. Surgery can remove cancer focuses but cannot do the same for the micro-focuses and neither can extinguish the free cancer cells that are often the origin of relapse. Chemotherapy with anticancer drugs is the main auxiliary treatment but often fails because of their toxic and side effects that are not endurable for the patients. Over the past few decades, the field of cancer biology has progressed at a phenomenal rate. However, despite astounding advances in fundamental cancer biology, these results have not been translated into comparable advances in clinics. Inadequacies in the ability to administer therapeutic agents with high selectivity and minimum side effects largely account for the discrepancies encompassing cancer therapies. Hence, considerable efforts are being directed to such a drug delivery system that selectively target the cancerous tissue with minimal damage to normal tissue outside of the cancer focuses. However, most of this research is still in the preclinical stage and the successful clinical implementation is still in a remote dream. The development of such a system is not dependent only on the identification of special biomarkers for neoplastic diseases but also on the constructing of a system for the biomarker-targeted delivery of therapeutic agents that avoid going into normal tissues, which remains a major challenge [1]. With the development of nanotechnology, few nanomaterial-based products have shown promise in the treatment of cancers and many have been approved for clinical research, such as nanoparticles, liposomes, and polymer-drug conjugates. The requirements for new drug delivery systems to improve the pharmacological profiles while decreasing the toxicological effects of the delivered drugs have also envisaged carbon nanotubes (CNTs) as one of the potential cargos for the cancer therapy. CNTs belong to the fullerene family of carbon allotropes with cylindrical shape. The unique physicochemical properties [2,3] of CNTs with easy surface modification have led to a surge in the number of publications in this interesting field. Apart from their uses in the cellular imaging with diagnostic effects in nanomedicine [4,5], CNTs are promising drug carriers in the target drug delivery systems for cancer therapies. Unlike other naocarriers, such as liposomes/micelles that emerged in the 1960s and nanoparticles/dendrimers that emerged in 1980s, it has emerged no more than 20 years for carbon nanotubes to be envisaged as target drug carriers. In this chapter, the works that have been carried out with CNTs in the field of cancer therapy are briefly introduced.

### Physicochemical properties of CNTs

**Figure 1:**
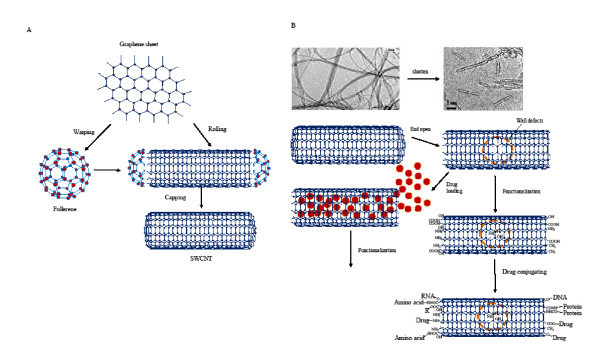
**The formation of SWCNT and its physical and chemical treatment for use as drug carriers**. (**A**) The schematic illustration of the structure formation of SWCNTs with the two ends closed. (**B**) The schematic illustration of the strategy for the preparation of the CNT-based drug delivery systems.

Carbon nanotubes are a huge cylindrical large molecules consisting of a hexagonal arrangement of sp^2 ^hybridized carbon atoms (C-C distance is about 1.4 Ǻ). The wall of CNTs is single or multiple layers of graphene sheets, of which those formed by rolling up of single sheet are called single-walled carbon nanotubes (SWCNTs) and those formed by rolling up of more than one sheets are called multi-walled CNTs (MWCNTs). Both SWCNTs and MWCNTs are capped at both ends of the tubes in a hemispherical arrangement of carbon networks called fullerenes warped up by the graphene sheet (Figure 1A). The interlayer separation of the graphene layers of MWCNTs is approximately 0.34 nm in average, each one forming an individual tube, with all the tubes having a larger outer diameter (2.5 to 100 nm) than SWCNTs (0.6 to 2.4 nm). SWCNTs have a better defined wall, whereas MWCNTs are more likely to have structural defects, resulting in a less stable nanostructure, yet they continue to be featured in many publications due to ease of processing. As for their use as drug carriers, there remain no conclusive advantages of SWCNTs relative to MWCNTs; the defined smaller diameter may be suitable for their quality control while the defects and less stable structure make their modification easier. CNTs vary significantly in length and diameter depending on the chosen synthetic procedure. SWCNTs and MWCNTs have strong tendency to bundle together in ropes as a consequence of attractive van der Waals forces. Bundles contain many nanotubes and can be considerably longer and wider than the original ones from which they are formed. This phenomenon could be of important toxicological significance [6,7]. CNTs exist in different forms depending upon the orientation of hexagons in the graphene sheet and possess a very high aspect ratio and large surface areas. The available surface area is dependent upon the length, diameter, and degree of bundling. Theoretically, discrete SWCNTs have special surface areas of approximately 1300 m^2^/g, whereas MWCNTs generally have special surface areas of a few hundred square meters per gram. The bundling of SWCNTs dramatically decreases the special surface area of most samples of SWCNT to approximately 300 m^2^/g or less, although this is still a very high value [8,9]. The markedly CNTs have various lengths from several hundreds of nanometers to several micrometers and can be shortened chemically or physically for their suitability for drug carriers (Figure 1B) [10] by making their two ends open with useful wall defects for intratube drug loading and chemical functionalization (Figure 1B).

### Functionalization of CNTs

**Figure 2:**
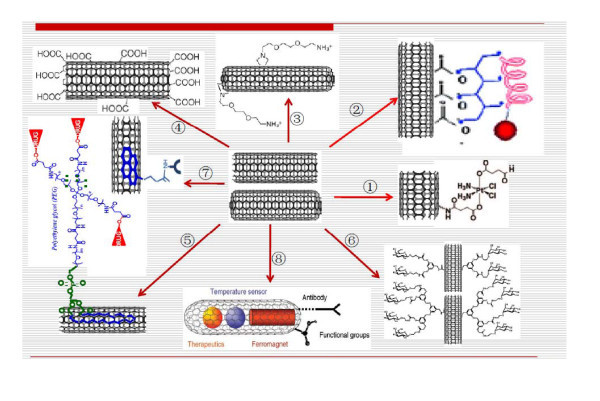
**The modification of CNTs**. Schematic illustration of modification of CNTs with various molecules. 1, Dhar et al. [70]; 2, Jia et al. [13]; 3, Georgakilas et al. 2002 [16]; 4, Peng et al. 1998; 5, Liu et al. [91]; 6, Gu et al. 2008; 7, Son et al. 2008; 8, Klingeler et al. 2009.

As drug carriers, the solubility of CNTs in aqueous solvent is a prerequisite for gastrointestinal absorption, blood transportation, secretion, and biocompatibility and so on; hence, CNT composites involved in therapeutic delivery system must meet this basic requirement. Similarly, it is important that such CNT dispersions should be uniform and stable in a sufficient degree, so as to obtain accurate concentration data. In this regard, the solubilization of pristine CNTs in aqueous solvents is one of the key obstacles in the way for them to be developed as practical drug carriers owing to the rather hydrophobic character of the graphene side walls, coupled with the strong π-π interactions between the individual tubes. These properties cause aggregation of CNTs into bundles. For the successful dispersion of CNTs, the medium should be capable of both wetting the hydrophobic tube surfaces and modifying the tube surfaces to decrease tube's bundle formation. To obtain desirable dispersion, Foldvari et al. have proposed four basic approaches [11]: (1) surfactant-assisted dispersion, (2) solvent dispersion, (3) functionalization of side walls, and (4) biomolecular dispersion. Among the above described approaches, functionalization has been the most effective approach. In addition, functionalization has been shown capable of decreasing cytotoxicity, improving biocompatibility, and giving opportunity to appendage molecules of drugs, proteins, or genes for the construction of delivery systems [12]. Up to now, there have been a lot of literatures on the functionalization of CNTs with various molecules (Figure 2A). The functionalization can be divided into two main subcategories: non-covalent functionalization and covalent functionalization (Figure 2B).

### Non-covalent functionalization

Many small, as well as large, polymeric anticancer agents can be adsorbed non-covalently onto the surface of pristine CNTs. Forces that govern such adsorption are the hydrophobic and π-π stacking interactions between the chains of the adsorbed molecules and the surface of CNTs. Since many anticancer drugs are hydrophobic in nature or have hydrophobic moieties, the hydrophobic forces are the main driving forces for the loading of such drugs into or onto CNTs. The presence of charge on the nanotube surface due to chemical treatment can enable the adsorption of the charged molecules through ionic interactions [13,14]. Aromatic molecules or the molecules with aromatic groups can be embarked on the debunching and solubilization of CNTs using nucleic acids and amphiphilic peptides based on the π-π stacking interactions between the CNT surface and aromatic bases/amino acids in the structural backbone of these functional biomolecules. Noncovalent functionalization of CNT is particularly attractive because it offers the possibility of attaching chemical handles without affecting the electronic network of the tubes.

Oxide surfaces modified with pyrene through π-π stacking interactions have been employed for the patterned assembly of single-walled carbon nanomaterials [15]. The carbon graphitic structure can be recognized by pyrene functional groups with distinct molecular properties. The interactions between bifunctional molecules (with amino and silane groups) and the hydroxyl groups on an oxide substrate can generate an amine-covered surface. This was followed by a coupling step where molecules with pyrene groups were allowed to react with amines. The patterned assembly of a single layer of SWCNT could be achieved through π-π stacking with the area covered with pyrenyl groups. Alkyl-modified iron oxide nanoparticles have been attached onto CNT by using pyrenecarboxylic acid derivative as chemical cross-linker [16]. The resulting material had an increased solubility in organic media due to the chemical functions of the inorganic nanoparticles.

Surfactants were initially involved as dispersing agents [17] in the purification protocols of raw carbon material. Then, surfactants were used to stabilize dispersions of CNT for spectroscopic characterization [18], optical limiting property studies, and compatibility enhancement of composite materials.

Functionalized nanotube surface can be achieved simply by exposing CNTs to vapors containing functionalization species that non-covalently bonds to the nanotube surface while providing chemically functional groups at the nanotube surface [19]. A stable functionalized nanotube surface can be obtained by exposing it to vapor stabilization species that reacts with the functionalization layer to form a stabilization layer against desorption from the nanotube surface while depositing chemically functional groups at the nanotube surface. The stabilized nanotube surface can be exposed further to at least another material layer precursor species that can deposit as a new layer of materials.

A patent [20] is pertinent to dispersions of CNTs in a host polymer or copolymer with delocalized electron orbitals, so that a dispersion interaction occurs between the host polymer or copolymer and the CNTs dispersed in that matrix. Such a dispersion interaction has advantageous results if the monomers of the host polymer/copolymer include an aromatic moiety, e.g., phenyl rings or their derivatives. It is claimed that dispersion force can be further enhanced if the aromatic moiety is naphthalenyl and anthracenyl. A new non-wrapping approach to functionalizing CNTs has been introduced by Chen et al. [21]. By this approach, the functionalization can be realized in organic and inorganic solvents. With a functionally conjugated polymer that includes functional groups, CNT surfaces can be functionalized in a non-wrapping or non-packaging fashion. Through further functionalization, various other desirable functional groups can be added to this conjugated polymer. This approach provided the possibility of further tailoring, even after functionalization. A process registered by Stoddart et al. [22] involves CNTs treated with poly{(5-alkoxy-m-phenylenevinylene)-co-[(2,5-dioctyloxy-p-phenylene) vinyl-ene]} (PAmPV) polymers and their derivatives for noncovalent functionalization of the nanotubes which increases solubility and enhances other properties of interest. Pseudorotaxanes are grafted along the walls of the nanotubes in a periodic fashion by wrapping of SWCNTs with these functionalized PAmPV polymers. Many biomolecules can interact with CNTs without producing of covalent conjugates. Proteins are an important class of substrates that possess high affinity with the graphitic network. Nanotube walls can adsorb proteins strongly on their external sides, and the products can be visualized clearly by microscopy techniques. Metallothionein proteins were adsorbed onto the surface of multi-walled CNT, as evidenced by high-resolution transmission electron microscopy (TEM) [23]. DNA strands have been reported by several groups to interact strongly with CNT to form stable hybrids effectively dispersed in aqueous solutions [24,25]. Kim et al. [26] reported the solubilization of nanotubes with amylose by using dimethyl sulfoxide/water mixtures. The polysaccharide adopts an interrupted loose helix structure in these media. The studies of the same group on the dispersion capability of pullulan and carboxymethyl amylase demonstrated that these substances could also solubilize CNTs but to a lesser extent than amylose. There are also some literatures that reported several other examples of helical wrapping of linear or branched polysaccharides around the surface of CNT [27].

### Covalent functionalization

Covalent functionalization gives the more secure conjunction of functional molecules. CNTs can be oxidased, giving CNTs hydrophilic groups as OH, COOH, and so on. Strong acid solution treatment can create defects in the side walls of CNTs, and the carboxylic acid groups are generated at the defect point, predominantly on the open ends. Excessive surface defects possibly change the electronic properties and cut longer CNTs into short ones. as drug carriers may need CNTs with different electronic properties and different lengths. In the preparation of some drug delivery systems, CNTs are deliberately cut into short pieces. The functional groups on the oxidized CNTs can further react with SOCl and carbodiimide to yield functional materials with great propensity for reacting with other compounds [28,29].

Covalent functionalization of SWCNTs using addition chemistry is believed to be very promising for CNT modification and derivatization. However, it is difficult to achieve complete control over the chemo- and region selectivity of such additions and require very special species such as arynes, carbenes, or halogens, and the reactions often occur only in extreme conditions for the formation of covalent bonds. Furthermore, the characterizatiuon of functionalized SWCNTs and the determination of the precise location and mode of addition are also very difficult [30]. The covalent chemistry of CNTs is not particularly rich with respect to variety chemical reactions to date. As regard to functionalization behavior of SWCNTs and MWCNTs, it has been reported that functionalization percentage of MWCNTs is lower than that of SWCNTs with the similar process [31], which is assumingly attributed to the larger outer diameter and sheathed nature of MWCNTs that render many of their sidewalls inaccessible; nonetheless, a comparative study on functionalizing single-walled and multi-walled carbon nanotubes is scarce hitherto in open literature.

In comparison with non-covalent functionalization, there are new substances to develop and therefore most patents regarding functionalization of CNTs registered to date are based on covalent chemistry. Though covalent procedures are not highly diverse yet, the end products vary exceedingly in terms of characteristics depending upon the incorporated species.

Methotrexate functionalization can be realized through 1,3-cycloaddition reaction [32]. Azomethine ylides consisting of a carbanion adjacent to an immonium ion are organic 1,3-dipoles, which give pyrrolidine intermediates upon cycloaddition to dipolarophiles. Through decarboxylation of immonium salts obtained from the condensation of α-amino acids with aldehydes or ketones, azomethine ylides can be easily produced. These compounds can make CNTs fused with pyrrolidine rings with varied substituents depending on the structure of used α-amino acids and aldehydes.

Using acyl peroxides can generate carbon-centered free radicals for functionalization of CNTs [33]. The promising method allows the chemical attachment of a variety of functional groups to the wall or end-cap of CNTs through covalent carbon bonds without destroying the wall or end-cap structure of CNTs [34], unlike in the case of treating with strong acid. Carbon-centered radicals generated from acyl peroxides can have terminal groups that render the modificated sites capable of further reaction with other compounds. For example, organic groups with terminal carboxylic acid functionality can further react with acyl chloride and an amine to form an amide or with a diamine to form an amide with terminal amine. The reactive functional groups attached to CNTs not only render solvent dispersibility improved but also offer reaction sites for monomers to incorporate in polymeric structures. Free radicals for functionalization can also be produced by organic sulfoxides. The key feature of this free radical method is its simplicity coupled with a reasonable choice of radical generating compounds [35].

A method for producing polymer/CNTs composites invented by Ford et al. [36] allows covalent attachments of polymers to CNTs. The resultant composites disperse in liquid media to form stable colloidal dispersions without separating for prolonged periods ranging from hours to months. The polymer functionalized CNTs are also capable of being dispersed into the parent polymer. The method has been effectively and conveniently used in the functionalization, solubilization, and purification of CNTs, although the stabilization of these dispersions is greatly dependent upon given colloidal systems.

A three-step method has been proposed by Barrera et al. [37], in which functionalized CNTs are used to prepare polymer composite in first place and then these CNTs are defuntionalized therein returning them to original chemistry. The first step is dispersing functionalized CNTs in a solvent to form a dispersion; the second is incorporating the dispersion of functionalized CNTs into a polymer host matrix to form a functionalized CNTs-polymer composite; and the third is modifying the functionalized CNTs-polymer composite with radiation, wherein the modifying comprises defunctionalization of the functionalized CNTs via radiation selected from the group consisting of protons, neutrons, alpha particles, heavy ions, cosmic radiation, etc. The feature of this method is that the functionalization is carried out only as assist dispersion, and CNTs are returned to its original characteristics after incorporating in polymer matrix.

A method to create new polymer/composite materials has been devised by Tour et al. [38] by blending derivatized carbon nanotubes into polymer matrices. Modification with suitable chemical groups using diazonium chemistry made CNTs chemically compatible with a polymer matrix, which allows the properties of CNTs to transfer to that of the product composite material as a whole. This method is simple and convenient. The reaction can be achieved by physical blending of derivatized CNTs with the polymeric material, no matter at ambient or elevated temperature. This method can be used in the fixation of functional groups to CNTs further covalently bonding to the matrix of host polymers or directly between two tubes themselves. Furthermore, CNTs can be derivatized with a functional group that is an active part of a polymerization process, resulting in a composite material in which CNTs are chemically involved as generator of polymer growth. This procedure ensures an excellent interaction between the matrix and CNTs since CNTs aid polymerization and growth of polymer chains that render them more compatible with the host polymer, although it does not address the question of CNT dispersion. Stanislaus et al. [39] functionalized the sidewalls of a plurality of CNTs with oxygen moieties. This procedure exposed CNT dispersion to an ozone/oxygen mixture to form a plurality of ozonized CNTs. The plurality of ozonized CNTs reacted with a cleaving agent to form a plurality of sidewall-functionalized CNTs.

As mentioned above, functionalization of CNTs can be achieved in acidic media [40]. Bundled CNTs can be separated as individual CNTs by dispersing them in an acidic medium, which exposes the sidewalls of CNTs, facilitating the functionalization. Once CNTs are dispersed in unbundled state, the functionalizing reaction occurs. This method is of great promising because it is easily scalable, providing for sidewall-functionalized CNTs in large, industrial quantities. In acidic medium, CNTs can be shortened, which causes loss of some properties of CNTs, but this shortening are sometimes needed for special purposes such as in the case of CNTs are used as oral drug carriers [10].

For studies on the use of CNTs in neurology at the nanometer scale, Mark et al. constructed an implant system [41], composed of CNTs and neurons growing from there. CNTs are functionalized with neuronal growth promoting agents selected from a group chemicals consisting of 4-hydroxynonenal, acetylcholine, dopamine, GABA (g-aminobutyric acid), glutamate, serotonin, somatostatin, nitrins, semaphorins, roundabout, calcium, etc. Functionalized CNTs in this system are employed for promoting the growth of neurons, which are clinically significant because it is possible to be used for effectively promoting nerve regeneration, bringing opportunity for stroke patients to recover from their paralyzed states.

CNTs have been demonstrated to be rather inert due to the seamless arrangement of hexagon rings without any dangling bonds in the sidewalls. The fullerene-like tips in the ends of the tubes are more reactive than the sidewalls. Various chemical reagents can react with the tips to attach chemical groups on them. However, it remains a challenge to realize the asymmetric functionalization of CNTs with each of their two endtips attached by different chemical reagents. The method of asymmetric end-functionalization has been tried by Dai and Lee [42] who employ physicochemical process to produce asymmetric end-functionalization of CNTs.

A method for functionalizing CNTs with organosilane species has been devised by Enrique et al. [43]. Hydroxyl-functionalized CNTs are prepared by reacting fluorinated CNTs with moieties comprising terminal hydroxyl groups and then to obtain organosilane-functionalized polymer-interacting CNTs by reacting the hydroxyl-functionalized CNTs with organofunctionalized silanol (hydrolyzed organoalkoxysilanes) bearing "polymer-interacting" functional moieties. Such CNTs can interact chemically with a polymer host material. This method has two benefits. The first is that the functionalized CNTs can provide strong attachment to both fiber (other CNTs) and matrix (polymer) via chemical bonds. With polymer compatible organofunctional silane, functionalized CNTs can be directly included into polymer matrices. The second is a high level of CNT unroping and the formation of relatively soluble materials in common organic solvents, offering opportunity for homogeneous dispersion in polymer matrices. Valery et al. [44], also invented a method regarding the functionalization of SWCNT sidewall through C-N bond substitution reactions with fluorinated SWCNTs (fluoronanotubes). Ford et al. patented a very convenient and simple method of solubilizing CNTs that involves mixing and heating of CNTs and urea to initiate a polymerization reaction of the isocyanic acid and/or cyanate ion to yield modified CNTs [45].

As a summary, there have been a lot of literatures and patents regarding the functionalization of CNTs. Of these techniques, most have not been used, but they are identical with those used in drug delivery systems. These functionalization methods provided candidate techniques, and there are great possibilities for them to be used in the construction of drug delivery systems in not too long a time. The functionalization of CNTs used in the construction of drug delivery systems will be discussed in later sections.

### *In vivo *behavior of functionalized CNTs

For all pharmaceuticals, precise determination of pharmacological parameters, such as the absorption, transportation, target delivery effects, blood circulation time, clearance half-life, organ biodistribution, and accumulation, are essential prerequisites for them to be developed into practically usable drugs [46]. For their drug carrier use, CNTs must be absorbed from the administration site into the body. There are quite a few ways for the administration of drugs, such as oral, vein injection, muscle injection, subcutaneous injection, and local injection and so on. The absorbed CNTs must be transported from the administration sites to the effects-related sites, such as cancer focuses, infection focuses, ischemia focuses, and so on. For the excretion, CNTs must be transported from everywhere in the body to the excretion organs such as kidney, liver, and so on. All of these questions must be made clear for the biosafety of CNTs used as drug carriers. Unfortunately, the data about these questions are still insufficient, although remarkable progress has been achieved.

### Administration, absorption, and transportation

As drug carriers, the administration, absorption, and transportation of CNTs must be considered for obtaining desired treatment effects. The studied ways of CNT administration include oral and injections such as subcutaneous injection, abdominal injection, and intravenous injection. There are different ways for the absorption and transportation when CNTs are administered in different ways. The absorbed CNTs are transported from the administration sites to the effects-relevant sites by blood or lymphatic circulation.

**Figure 3:**
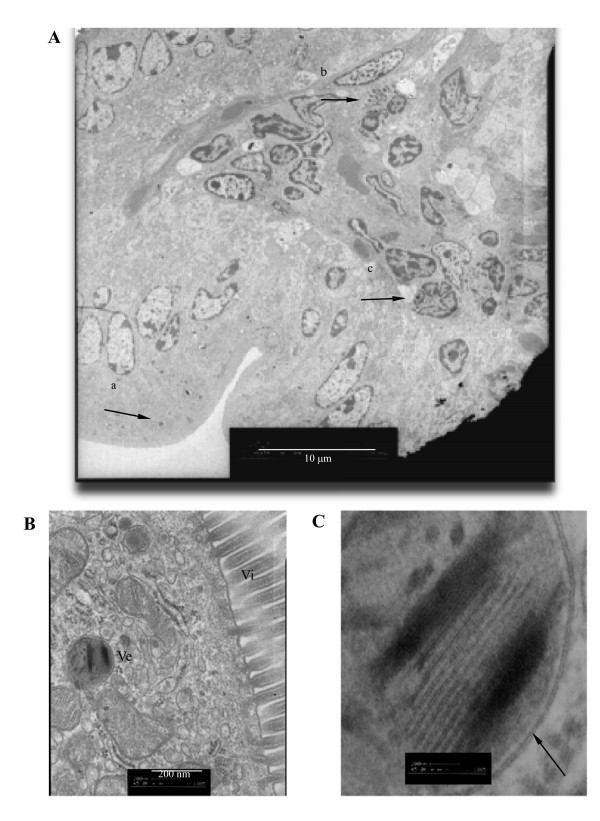
**The absorption of SWCNTs through intestinal columnar epithelial cells **[10]. (**A**) SWCNTs (arrows) found in the intestinal mucous membrane. (**B**) Magnification of the cell indicated by the left arrow in (A). Ve, transportion vehicles; Vi, villus of the columnar cells. (**C**) Magnification of the Ve, which has membrane with double lipid layers (arrow).

After administration, absorption is the first key step for drug carriers to complete their drug-delivering mission. However, there have been very few literatures on the absorption of CNTs from their administration sites. Yukako et al. studied the absorption of erythropoietin (EPO) loaded in CNTs from rat small intestine and the effect of fiber length on it. Erythropoietin-loaded carbon nanotubes (CNTs) with surfactant as an absorption enhancer were prepared for the oral delivery of EPO using two types of CNTs, long and short fiber length CNTs. The results of ELISA measurements revealed that serum EPO level reached to *C*_max_, 69.0 ± 3.9 mIU/ml, at 3.5 ± 0.1 h, and the area under the curve (AUC) was 175.7 ± 13.8 mIU h/ml in free EPO group, which was approximately half of that obtained with that loaded into short fiber length CNTs, of which *C*_max _was 143.1 ± 15.2 mIU/ml and AUC was 256.3 ± 9.7 mIU h/ml [47]. When amphoteric surfactant, lipomin LA, sodium β-alkylaminopropionic acid, was used to accelerate the disaggregation of long fiber length CNTs, *C*_max _was 36.0 ± 4.9 and AUC was 96.9 ± 11.9, showing less bioavailability of EPO. These results suggest that CNTs themselves are capable of being absorbed and that the short fiber length CNTs deliver more both EPO and absorption enhancer to the absorptive cells of the rat small intestine and the aggregation of CNTs is not the critical factor for the oral delivery of EPO. Our recent works further demonstrated that the physically shortened CNTs orally administered can be absorbed through the columnar cells of intestinal mucous membrane, which was confirmed by transmission electron microscope (Figure 3) [10]. In the experiment, high-speed shearing-shortened SWCNTs were used. The absorb ability of intestinal tract for CNTs is of great significance because this makes it possible to develop oral drug delivery systems based on CNTs.

When subcutaneously and abdomenally administered, a part of CNTs exist persistently in the local tissues while some of them may be absorbed through lymphatic canal. Because the fenestra in the endothelial cells of blood capillaries are 30 nm to approximately 50 nm while that in the endothelial cells of lymphatic capillaries are larger than 100 nm in diameter, the lymph absorption of bundled CNTs seemed to be easier than blood absorption. The lymphatically absorbed CNTs migrate along the lymph canal and are accumulated in the lymph node, which is in fact a lymphatic target effects. This is clinically important because lymphatic metastasis occurs extensively in cancers, resulting in frequent tumor recurrence, even after extended lymph node dissection. If anticancer drugs are loaded into CNTs, they will be delivered into lymph system, where the drugs will be released to kill metastatic cancer cells. Ji et al. successfully delivered gemcitabine to lymph nodes with high efficiency by using lymphatic targeted drug delivery system based on magnetic MWCNTs under the magnetic field guidance [48,49]. The result suggests that the anticancer drug delivery system based on CNTs is advantageous over the current ways to deliver chemotherapeutic agents to lymph nodes. In another approach [50], water-soluble MWCNTs were subcutaneously injected into the left rear foot pad of rat; the biopsy found that the accumulation of MWCNTs in left popliteal lymph nodes was more obvious than in other regions, and micropathology revealed large MWCNT collections in the popliteal lymph nodes. At the same time, the biopsy experiments found no presence of MWCNTs in the major internal organs such as liver, kidney, and lung, which suggests the properties of MWCNT lymphatic targets.

When administered through veins, CNTs can directly get into blood circulation and distribute in many internal organs, such as liver, spleen, heart and kidney (unpublished date). Some studies demonstrated that the blood clearance of intravenously injected CNTs largely depends upon the surface modification. Singh et al. found that, following intravenous administration, ^111^In-labeled water-soluble SWCNTs functionalized with diethylenetriaminepenta acetic acid (average diameter, 1 nm; average length, approximately 300 to 1,000 nm) can be eliminated rapidly from blood in the form of intact CNT molecules, displaying a half-life of 3 h, with no specific organ accumulation [51]. In a recent literature, it was found that the clearance of 1,4,7,10-tetraazacyclododecane-1,4,7,10-tetra-acetic acid functionalized CNTs complexed with yttrium-86 or ^111^In and anti-CD20 antibody rituximab for targeting to malignant B cells was rapid, although the blood half-lives have not been reported [52].

Polyethylene glycol(PEG)ylation is believed to be one of the most important strategies to prolong the circulation time of CNTs in blood because the surface coverage with PEG lowers the immunogenicity of the carriers and prevents their nonspecific phagocytosis by the reticuloendothelial system (RES); thus, their half-life in blood circulation is prolonged. In fact, it has been found that PEGylated CNTs can persistently exist within liver and spleen macrophages for 4 months with excellent compatibility [53]. In a recent investigation, it was observed that fluorescein isothiocyanate (FITC)-labeled PEGylated SWCNTs can penetrate the nuclear membrane and get into nucleus in an energy-independent way [54]. The presence of FITC-PEG-SWCNTs in nucleus did not produce any significant ultrastructural change in the nuclear organization and had no significant effects on the growth kinetics and cell cycle distribution for up to 5 days. Surprisingly, upon removal of the FITC-PEG-SWCNTs from the culture medium, the internalized FITC-PEG-SWCNTs rapidly moved out of the nucleus and were released from the cells, suggesting that the internalization of CNTs into and excretion of CNTs from the cells are a bidirectional reversible process. These results illustrated well the successful exploitation of SWCNTs as ideal nanovectors for biomedical and pharmaceutical applications and, they will drive the concern about the excretion problems out of people's heart.

### Distribution

Distribution indicates the sites or places the absorbed CNTs can arrive and exist there, which are of great importance in clinical pharmacology and toxicology of CNTs as drug carriers.

There have been experiments to investigate *in vivo *and *ex vivo *biodistributions, as well as tumor targeting ability of radiolabeled SWCNTs (diameter, approximately 1 to 5 nm; length, approximately 100 to 300 nm) noncovalently functionalized with phospholipids(PL)-PEG in mice using positron emission tomography and Raman spectroscopy, respectively. It was interesting to note that the PEG chain lengths determine the biodistribution and circulation of CNTs. PEG-5400-modified SWCNTs have a circulation time (*t*_1/2 _= 2 h) much longer than that of PEG-2000-modified counterpart (*t*_1/2 _= 0.5 h), which may be attributed to the lower uptake of the former by the RES as compared with that of the later. By further functionalization of these PEGylated SWCNTs with arginine-glycine-aspartic acid (RGD) peptide, the accumulation in integrin-positive U87MG tumors was significantly improved from approximately 3% to 4% to approximately 10% to 15% of the total injected dose (ID)/g, owing to the specific RGD-integrin a_v_b_3 _recognition. Raman signatures of SWCNTs were further used to directly probe the presence of CNTs in mice tissues and confirmed the radiolabel-based results [55]. In another experiment to evaluate the influences of PEG chain lengths on cellular uptake of PEGylated SWCNTs, it has been found that adsorbing shorter chain PEG (PL-PEG-2000) to SWCNTs was incapable of protecting CNTs from macrophagocytosis both *in vitro *and *in vivo*, while adsorbing longer chain PEG (PL-PEG-5000) effectively reduced their nonspecific uptake of CNTs *in vivo *[56]. Functionalization of SWCNTs with PEG grafted branched polymers, namely poly(maleicanhydride-alt-1-octadecene)-PEG methyl ethers (PMHC18-mPEG) and poly (g-glutamic acid)-pyrine(30%)-PEG methylethers (70%) (gPGA-Py-mPEG), the blood circulation time was remarkably prolonged (half-life of 22.1 h for gPGA-Py-mPEG and 18.9 h for MHC18-mPEG) after intravenous injection into mice [57]. Further research reveals that the tumor accumulation of PEG-SWCNTs was 8% ID/g and 9% ID/g of the intravenously administered doses in EMF6 model (breast cancer in BABL/c mice) and the Lewis model (lung cancer in C57BL mice), respectively. SWCNTs covalently modified with PEG showed longer half-life in blood circulation in comparison with those noncovalently modified with PEG of similar chain lengths. SWCNTs covalently conjugated with branched chains of 7-kDa PEG effectively increased the half-life of SWCNTs up to 1 day, which is the longest among all of the tested PEGs. And this length chain PEG-modified SWCNTs had near-complete clearance from the main organs in approximately 2 months. There seemed to be a length limits in the relations between PEG chain lengths and their effects to increase the blood circulation time. Further increase in molecular weight from 7 to 12 kDa had no influence on the blood circulation time and RES uptake [58].

There are few literatures on the *in vivo *biodistribution properties of radionuclide-filled CNTs, although they have been extensively used as drug delivery systems or radiotracers. A very recent study revealed that surface functionalization of ^125^I-filled SWCNTs offers versatility towards modulation of biodistribution of these radio-emitting crystals, in a manner determined by the system that delivers them, which gave great promises for the development of organ-based therapeutics [59]. Nanoencapsulation of iodide within SWCNTs facilitated its biodistribution in tissues, and SWCNTs was completely redirected from tissue with intrinsic affinity (thyroid) to lungs. In this experiment, Na^125^I-filled glyco-SWCNTs were intravenously administered into mice and tracked *in vivo *by single photon emission computed tomography. Tissue-specific accumulation (lung in this case), coupled with high *in vivo *stability, prevented excretion or leakage of radionuclide to other high-affinity organs (thyroid/stomach), allowing ultrasensitive imaging and delivery of unprecedented radiodose density [60].

### Metabolism and excretion

The nonbiodegradability in the body and non-eliminatability from the body interrogate on the possibility of their successful use in clinical practice, which has been always concerned about.

Functionalized SWCNTs seem to be metabolizable in animal body. For example, SWCNTs with carboxylated surfaces have demonstrated their unique ability to undergo 90-day degradation in a phagolysosomal simulant, resulting in shortening of length and accumulation of ultrafine solid carbonaceous debris. Unmodified, ozonolyzed, aryl-sulfonated SWCNTs exhibit no degradation under similar conditions. The observed metabolism phenomenon may be accredited to the unique chemistry of acid carboxylation, which, in addition to introducing the reactive, modifiable COOH groups on CNT surfaces, also induces a collateral damage to the tubular graphenic backbone in the form of neighboring active sites that provide points of attack for further oxidative degradation [59]. Some experiments demonstrated that CNTs persisted inside cells for up to 5 months after administration; short (< 300 nm) and well-dispersed SWCNTs effectively managed to escape the RES and finally were excreted through the kidneys and bile ducts [61].

A very recent investigation reveals that the biodegradation of SWCNTs can be catalyzed by hypochlorite and reactive radical intermediates of the human neutrophil enzyme myeloperoxidase in neutrophils. The phenomenon of CNT metabolism can also be seen in macrophages to a lesser degree. Molecular modeling further reveals that the interaction between basic amino acid residues on the enzyme backbone and carboxyl acid groups of CNTs is favorable to orient the nanotubes close to the catalytic site. Notably, when aspirated into the lungs of mice, the biodegradation of the nanotubes does not engender an inflammatory response. These findings imply that the biodegradation of CNTs may be a key determinant of the degree and severity of the inflammatory responses in individuals exposed to them. However, further studies are still required in order to draw an appropriate conclusion [62].

### CNT-based drug delivery

While attachment of drugs to suitable carriers significantly improves their bioavailability, owing to their increased residence time in blood circulation and enhanced solubility, the therapeutic efficacy of the drug can be improved by the site-selective accumulation in the pathological zone of interest that sometimes were called therapeutic-effects-related sites. The unique capability of CNTs to penetrate cell membranes paves the road for using them as carriers to deliver therapeutic agents into the cytoplasm and, in many cases, into the nucleus. The intrinsic spectroscopic properties of CNTs, such as Raman and photoluminescence, afford additional advantages for tracking and real-time monitoring of drug delivery efficacy *in vivo*.

### Intracellular drug delivery

To study the cellular drug delivery, *in vitro *experiments have unique advantages, which are convenient to carry out; experiment conditions are easy to control and can give reliable results, although they cannot completely represent *in vivo *case.

#### Small molecules

Most of the anticancer agents are small molecules and can be loaded into or onto CNTs either by physical adsorption through p-p stacking interactions between pseudoaromatic double bonds of the graphene sheet and the drug molecules, or covalent immobilization of the interest drug molecules onto the reactive functional groups present on the sidewalls of CNTs.

Recently, Borowiak-Palen et al. reported that cisplatin, a small molecule, can be loaded into SWCNTs with a diameter of 1.3 to 1.6 nm [63]. The cisplatin incorporated into the tubes was proved with Raman spectroscopy, infrared spectroscopy, and high-resolution transmission electron microscopy (TEM). Drug-release study using dialysis membrane method revealed that cisplatin was continually released for almost a week, with maximum release during 72 h and up to 1 week. The encapsulation was 21 μg of drug per 100 μg of SWCNTs as revealed by thermogravimetric analysis. Cytotoxicity studies carried out on DU145 and PC3 human prostate cancer cell lines using 3-(4,5)-dimethylthiahiazo (-z-y1)-3,5-di- phenytetrazoliumromide (MTT) cell proliferation assay showed that the cell viability decreased with an increase in the concentration of the CNT-based nanovector, whereas blank CNTs showed no significant effects. Computational methods revealed the feasibility of interactions between CNTs and drug molecules [64]. For cisplatin acceptance or incorporation, CNTs must have a radius of at least 4.785 Å (0.4785 nm). In fact, most of the experimentally used CNTs have diameters greater than 4.785 Å. So, it is inferred that cisplatin is likely to be encapsulated inside the nanotubes [65].

Doxorubicin can be loaded on CNT to form supramolecular complexes based on p-p stacking interactions by simply mixing the drug with an aqueous dispersion of CNTs stabilized by Pluronic F127 (nonionic surfactant). The doxorubicin loading on MWCNTs was observed by measuring the emission spectrum of doxorubicin via fluorescence spectrophotometry. With the increase in the concentration of MWCNTs from 5 to 20 μg/ml, the fluorescence intensity of doxorubicin dramatically decreased with the final concentration in the suspension remaining constant (10 μg/ml), a part of which is attributed to the quenching of fluorescence. It was found that a mass ratio of 1:2 is optimum for maximum interaction/quenching ratio. TEM structural characterization revealed that CNTs present as well-individualized, dispersed nanotubes, confirming the polymer molecules' ability to disperse the CNTs effectively. On MCF-7 human breast cancer cell line, it was revealed that the doxorubicin-MWCNT complex shows enhanced cytotoxity in comparison with both doxorubicin alone and doxorubicin-Pluronic complexes. The enhanced cytotoxicity obtained with the doxorubicin-MWCNT complex indicates that MWCNTs can effectively enhance the delivery of doxorubicin and hence improve the cellular uptake of the drug [66], although *in vivo *studies are essential in order to further validate the efficiency of the reported system. Some other groups have also developed doxorubicin-loaded nanotubes but with more complex system structures. The system was composed of oxidized SWCNTs trifunctionalized with doxorubicin, a monoclonal antibody (mAb), and a fluorescent marker and therefore can be used for targeting, imaging, and therapeutic effects simultaneously. Confocal microscopy observation revealed that the complex was efficiently taken up by cancer cells and the doxorubicin was released subsequently and then translocated to the nucleus, while SWCNTs remain in the cytoplasm [67]. Of course, such a complex system requires rigorously investigating in order to check the integrity of the SWCNT hybrids during the course through biological milieu for its *in vivo *use. Zhang et al. have successfully prepared biocompatible and water-dispersible multifunctional drug delivery system with doxorubicin-loaded polysaccharide functionalized SWCNTs, which presents stimuli-responsive drug-release characteristics in addition to simultaneous targeting. The chitosan/alginate polymer chains have been wrapped around the CNTs simply by sonication and stirring of a chitosan/alginate solution containing CNTs. At acidic pH, hydrophilicity of doxorubicin is enhanced, which facilitates its detachment from the CNT surface. Compared with normal tissues, physiological pH condition of tumor environment and intracellular lysosomes is more acidic, and therefore, this system seemed to be able to intelligently release drugs in tumor tissues. Through tethering the free amino groups of chitosan with folic acid (FA), the targeting effects may be further improved. Such nanocarrier-based drug delivery systems for doxorubicin could be more selective and effective than the free drug and have the promise to result in reduced toxicity and side effects in patients, along with a smaller drug dose needed for chemotherapy [68]. Thus, such CNT-based supramolecular systems with structural uniqueness of the doxorubicin are capable of self-targeting due to the aforementioned mechanisms. Furthermore, this can be bolstered through ligand-based targeting by incorporation of special ligands, similar to RGD peptide, which targets integrin receptors, making it a multitargeted modality complementing each other for selective action at cancerous tissue [69].

Antioxidants have been considered to play a significant role in cancer therapy owing to their ability to combat oxidative stress. However, their poor solubility mitigates the reaping of the benefits from these compounds. This drives us to bring them under the canopy of nanocarriers in order to use them as practical pharmaceuticals. Covalently PEGylated ultrashort SWCNTs can be linked to the antioxidant, amino butylated hydroxy toluene, through ionic interactions by simple stirring of the mixture. Residual carboxylic acid groups on the oxidized CNT allow the ionic interactions with the amine group of the butylated hydroxy toluene. The formulation was evaluated using an oxygen radical absorbance capacity assay, in which a fluorescent probe's loss of fluorescent intensity is monitored in the presence of oxygen radicals. Oxygen-radical scavengers can keep the fluorescence of the probes. The fluorescence intensity remains unaffected until the radical scavenger is consumed when oxygen-radical scavengers are added to the system. The assay readout can be compared to the radical scavenging ability of a known radical scavenger, Trolox, a vitamin E derivative. The radical scavenging ability of the composite was found to be as high as 1,240 times that of Trolox. However, when the butylated hydroxy toluene functionalization was carried out through covalent addition to the sidewall, the antioxidant activity of the system was found to be decreased [14], suggesting that not all functionalizations are beneficial for antioxidant activity.

Poor blood circulation times of platinum anticancer drugs result in insufficient uptake by tumor tissues and intracellular DNA binding due to their unusually low size, making them suitable candidates for a nanoparticle-based drug delivery system to improve their pharmacological performance. For this purpose, a "longboat delivery system" has been prepared for the platinum warhead. In this system, a platinum complex [Pt (NH_3_)_2_Cl_2_(O_2_CCH_2_CH_2_CO_2_H) (O_2_CCH_2_CH_2_CONH-PEG-FA) derivatized with PEG and folate (FA) was attached to the surface of SWCNT functionalized with amino groups (SWNT-PL-PEG-NH_2_) through multiple amide linkages. Such a unique surface design facilitates active targeting of the prodrug to the tumor cell, where cisplatin is released upon intracellular reduction of Pt(IV) to Pt(II) after endocytosis. Internalization studies revealed not only high and specific binding of the SWCNT-tethered conjugate to the folate receptor but also many fold enhancement in activity (by a factor of 8.6) in comparison with free cisplatin [70]. A similar kind of SWCNT conjugate without the targeting moiety showed 2.5 times more toxic on NTera-2 cells [71].

There are quite a few literatures on the attempts to solve CNTs through polymers such as PL-PEG and chitosan among other polymers and then functionalizing them with drugs/ligands. However, Murakami et al. have demonstrated a more novel approach of solving carbon nanohorns through doxorubicin-PEG conjugates. The stacking interactions between the nanohorns and doxorubicin aid indirect attachment of PEG to carbon nanohorns for the enhancement of their dispersibility. This approach is also true for CNTs [72].

Polymeric drug conjugates, as a new class of systems, have been envisaged for tumor tissue-specific delivery of anticancer drugs [73]. Accumulation within tumor tissues can be achieved by the macromolecular size of polymeric drug conjugates, which enables them selective for cancerous tissues because of enhanced permeability and retention (EPR) effects. The pathophysiologic factors of the tumor cells, such as EPR, poor venous and lymph drainage, acidic pH, and relatively high temperature, improve the pharmacological performance of polymeric-based systems. On the same lines, CNT conjugates are being explored for improving cancer therapy, although it is still a serious challenge. Methotrexate (MTX) is a drug widely used against cancer; however, it suffers from low cellular uptake. Conjugation of MTX to CNTs enhances its internalization via the functionalized CNTs, representing a promising approach to overcome its limited cellular uptake. Two orthogonally protected amino groups were conjugate onto the side walls of CNTs and subsequently derivatized with FITC and MTX using the 1,3-dipolar cycloaddition of azomethine ylides. Epifluorescence and confocal microscopy studies suggested that MTX was rapidly internalized by CNTs and drug efficacy was enhanced [69]. Magnetic CNTs complexed with a layer of magnetite (Fe_3_O_4_) nanoparticles on the inner surface of the nanotubes have been used for lymphatic tumor targeting. Through nanoprecipitation, PL-PEG-FA functionalized magnetic CNTs can be impregnated with chemotherapeutic agents, such as 5-fluorouracil and cisplatin. Such a system can be guided by an externally placed magnet to target regional lymphatic nodes [74]. Although this is a little complex, the procedure seems to have practical significance.

Dendrimers, synthetic macromolecules, have tree-like and well-defined branch unique features, such as a multivalent surface (nanoscaffolding), interior shells, and a core to which the dendrons are attached, showing great promise for them to be used as drug carriers [75]. Shi et al. have tried to integrate the properties of CNT with that of dendrimers. MWCNTs were functionalized with generation 5 (G5) amine-terminated polyamidoamine dendrimers, on which FITC and folic acids were covalently linked. It was found that the nanocomposites are stable and biocompatible. Through amide linkage using 1-ethyl-3-(3-dimethylaminopropyl) carbodiimide (EDC) chemistry, the dendrimers were attached to the COOH groups present on oxidized CNTs. *In vitro *experiments demonstrated that the MWCNTs functionalized with folate have selectively target effects of the cancer cells that overexpress folate receptors. The integration of dendrimers with CNTs provided multivalent amine-rich periphery for the combination of drug molecules with CNT surfaces, greatly improving the effective therapeutic payload by incorporating drug molecules into dendrimer cavity [76].

#### Proteins

CNTs not only can deliver drugs of small molecules but also can deliver proteins. MWCNTs have been used as cellular carriers of recombined ricin A chain protein toxin (RAT) for tumor targeting. The complexes of RAT and MWCNT were capable of translocating in the cytoplasm of various cell lines, including L-929, HL7702, MCF-7, HeLa, and COS-7, and showed excellent performance of their biological functions, as evidenced by the effects of inducing cell apoptosis or death. In comparison with RTA alone, MWCNT-RTA conjugates achieved three times higher cell death rates for L-929, HL7702, MCF-7, HeLa (75% mortality), and COS-7 cells. Coupling of HER2 to MWCNTs-RTA complexes caused selective recognition of HER2/neu receptor [77].

To improve the efficacy of breast cancer targeting and therapy, anti-HER2 IgY antibodies were covalently coupled to the side walls of SWCNTs using EDC chemistry. Single-cell level Raman spectroscopic observation demonstrated that signals collected from the SK-BR-3 cells treated with the targeted nanoconjugate were significantly greater than that from the control cells. Near-infrared (NIR) irradiation showed selectively destructive effects on the HER2-expressing SK-BR-3 cells while no harming effects on HER2-free MCF-7 cells. There were also cells thermally ablated without the internalization of SWCNTs as observed through confocal microscopy, which may be attributed to the sharp local temperature increase [78]. Tumor-targeting CNT constructs were synthesized by McDevitt et al. from a water-soluble precursor CNTs functionalized with covalently conjugating multiple copies of tumor-specific mAbs, radiometal-ion chelates, and fluorescent probes. They demonstrated that the nanoconstructs were selectively reactive with human cancer cells. The experiments were designed to observe the target effects in a model of disseminated human lymphoma and in cells by flow cytometry and cell-based immunoreactivity assays versus appropriate control cells. Chakravarty et al. in a pioneering study, used biotinylated polar lipids (1,2-distearoyl sn-glycero-3-phosphoethanolamine-*N*-[biotinyl (PEG)2000] [DSPE-PEG(2000)-biotin]) to prepare stable, biocompatible, noncytotoxic CNT dispersions. Then, CNTs were functionalized by one of two different neutralite avidin-derivatized mAbs against either human CD22 or CD25. The peripheral blood mononuclear cells activated by CD22^+^CD25^- ^and CD22^-^CD25^+ ^cells can be bound only by the CNTs bearing the corresponding mAbs, respectively. And only the cells bound by the corresponding nanosystems were ablated after exposure to NIR light [79]. Kam et al. reported an approach similar to that of Chakravarty et al., in which targeting effects were achieved by using PL-PEG-FA functionalized SWCNTs [80].

Tumor lysate proteins (TLP) are composed of various tumor proteins that develop in a large number of tumors and patients, irrespective of the genetic origins of tumors. However, defined tumor markers, such as target antigens, are still in a lack, rendering the antibody responses difficult to assess, and since there is a lack of the nature of the immunogens, the efficacy of therapy involving the lysate proteins was generally assessed by tumor rejection, tumor growth retardation, or prolonged survival of the immunized mice rather than by antibody production. The anticancer immune response of TLP against multiple tumors [81] has been improved by CNTs. The efficacy of the RGD peptide for targeting tumors with overexpressed a_v_b_3 _integrin receptor is well established. An application example has exploited a similar strategy for active targeting of RGD-functionalized, PEGylated CNTs to integrin-positive tumors in mice. Based on similar lines, a_v_b_3 _mAbs were used to target the PL-PEG-modified SWCNTs. As targeting ligands, a_v_b_3 _mAbs have their advantages in terms of their high specificity towards antigen a_v_b_3 _and greater stability *in vivo *relative to RGD peptide. In spite of cell culture-based studies in U87MG (human glioblastoma cancer cells) and MCF-7 (human breast cancer cells) revealing good targeting efficiency, the behavior of such a macromolecular structure needs to be traced inside the complex biological system to confirm its value in cancer therapy [82].

#### RNA, DNA, or genes

The application of CNTs as gene carriers in gene delivery has been considered quite promising. Gene therapy involves not only the gene-based treatment for cancers but also that for the infectious diseases by introducing genetic materials. It is generally believed that the tumor formation is the results of the gene alterations and gene therapy aims to correct them. For all of the gene-based therapeutic strategies, efficient and safe gene delivery systems have become imperative to develop, especially the gene vectors because it is relatively easy to obtain corresponding genes. There have been two subcategories of gene vectors including many viral and nonviral vectors. Viral vectors have been modified to eliminate their toxicity and maintain their high gene transfer capability. However, their limited capacity for transgenic materials and safety, particularly immunogenicity, has compelled researchers to increasingly shift attention upon nonviral vectors as an alternative. Nonviral vectors are mainly based on cationic polymers. It is just recent thing that CNTs emerge as DNA carriers owing to their unique physical, chemical, and biological properties [83].

Charged hybrid DNA/SWCNT complexes can be obtained by sonicating the suspensions composed of single-stranded DNA and CNTs. The aromatic nucleobases are believed to bind to the graphene side walls through π-π stacking effects. DNA molecules can be confined and oriented by CNTs that acted as scaffolds, which were wrapped around by DNA macromolecules. Moreover, there are different interaction energies with nanotubes for different nucleobases. The sugar and phosphate groups remain at the periphery relative to the bases, playing the roles of enhancing the dispersibility of CNTs. The spontaneous wrapping of DNA around nanotubes has been also confirmed by atomic-force microscopy and spectroscopic studies. Such a system can definitely prove useful in gene delivery. Several experimental studies in this area [84] were discussed here.

A material capable of binding negatively charged siRNA through electrostatic interactions can be obtained by functionalizing SWCNTs with hexamethylenediamine (HMDA) and poly(diallyldimethyl ammonium chloride) (PDDA). PDDA was bound by noncovalent interactions, whereas HMDA was covalently linked to the oxidized CNTs. The experiments on isolated rat heart cells revealed that the effect of *ERK1*/*ERK2 *genes loaded onto HMDA-PDDA-SWCNTs enhanced efficiency for *ERK1 *and *ERK2 *by a factor of nearly 80%. Furthermore, the biocompatibility has also been improved. No significant cytotoxicity was caused by PDDA-HMDA-SWCNT complexes at concentrations of 10 mg/l [85]. Pantarotto et al. used CNTs functionalized with ammonium as vectors for transfecting plasmid (b-galactosidase encoded gene, b-gal) into HeLa and CHO cell lines. The transfection efficiency was calculated based on the experimental data to be proportional to the CNT/DNA charge ratio, with the highest at 6:1. In comparison with DNA alone, transfection efficacy of CNT-DNA conjugates was ten times higher [86]. Through layer-by-layer electrostatic self-assembly of polycationic agents such as polyethylenimine (PEI), PDDA, polyamidoamine dendrimers, and chitosan on nanotube side walls, cationic polyelectrolyte functionalization of CNTs can be obtained, which are considered to be effective vehicles for gene delivery [87]. For example, green fluorescence protein (EGFP) reporter protein expression has been enhanced with functionalized CNTs complexed with nanochitosan as the gene carriers for the delivery of encoding DNA. In comparison with blank chitosan, CNT-chitosan nanocomplexes have exhibited significantly higher transfection efficiency [88]. The feasibility of bifunctional, MWCNT-quantum dot-based hybrids as gene transfer vector systems have also been explored by recent investigations. Mercaptoacetic acid-capped CdTe quantum dots, as fluorescent probes, were linked to antisense oligodeoxynucleotides to suppress telomerase expression. Their activity is unusually high in 90% of cancer cells compared with normal cells. Then, these complexes further complexed with functionalized MWCNTs through ionic interactions. Based on confocal and flow-cytometric studies, efficient intracellular transporting, strong cell nucleus localization, and high delivery efficiency of antisense oligodeoxynucleotides were exhibited by this system in comparison with free antisense oligodeoxynucleotides in HeLa cells. When the *EGFP *gene transfected using PEI-MWCNT as gene vectors, EGFP was overexpressed, further confirming the role played by the nanostructures. The nanosystems and the enhanced proton sponge effects of PEI coating on the surface of MWCNTs prevent DNA from enzyme degradation [13] in cytoplasm and increase the ratio of the gene to be expressed afterwards. However, efficacy of such delivery systems was critically dependent on the chemistry of surface functionalization. Kam et al. have demonstrated that effective transporting, enzymatic cleaving, and releasing of DNA from SWCNT transporters and subsequent nuclear translocation of DNA oligonucleotides in mammalian cells can also be achieved by incorporating cleavable disulfide bonds on the surface of PL-PEG functionalized SWCNTs. Such functionalization showed not only that the delivery of siRNA was highly efficient but also that the RNAi functionality achieved in this way was more potent than the conventional transfection agents that have been widely used [89].

### *In vivo *studies on drug delivery

As drug carriers, they will be finally used in living animals and human. Although the results of the *in vitro *experiments have provided a lot of useful information about the application of CNTs as drug carriers, only the *in vivo *experiments can give corroboration for the usefulness of CNTs in practical gene delivery for cancer therapies. However, there have been only limited *in vivo *studies reported on the application of CNTs as the molecular transporter in drug delivery.

#### Drug delivery targeted to lymphatic system

Many cancers metastasize through lymphatic canal. The drug delivery systems targeted to the lymphatic system can block the metastasis of cancers effectively. Using radical polymerization, polyacrylic acid (PAA) can be appended onto CNTs, making them highly hydrophilic. Through coprecipitation, Fe_3_O_4_-based magnetic nanoparticles can be adsorbed on the PAA-CNT surface. Through the interaction with COOH groups of grafted PAA, the nanoparticles can be stabilized from clustering. By stirring the solution containing PAA-CNT, Fe_3_O_4_-based magnetic nanoparticles, and gemcitabine for 24 h, gemcitabine was loaded into the nanosystem with a loading efficiency of 62%. It was found that CNTs were seen only in the local lymphatic nodes and were absent in the major organs, such as liver, kidney, heart, spleen, and lungs, after 3 h of subcutaneous injection. Without the help of such a nanostructures [90], gemcitabine cannot preferentially distribute in the lymphatic system.

#### Drug delivery targeted to tumor

To deliver anticancer drugs into cancer focus is the prerequisite for the drugs to develop their effects. However, some drugs cannot arrive or enter cancer tissues because of their short residence time in blood circulation. For example, the efficacy of paclitaxel (PTX), a widely used chemotherapeutic agent in cancer therapy, is often limited by its poor solubility in aqueous medium and nonspecific cytotoxicity, thereby preventing it from efficiently reaching the cancer focuses. Furthermore, the solubilizer cremophor in current formulation (Taxol) has exhibited allergenic activity, prompting the search for alternative delivery systems. For this purpose, Liu et al. successfully conjugated PTX to branched PEG chains on SWCNTs via a cleavable ester bond to obtain a water-soluble SWCNT-PTX complex. In a murine 4T1 breast cancer model, the SWCNT-PTX complex showed an efficacy higher than that of the clinically used Taxol in suppressing tumor growth. The blood circulation time almost was sixfold higher than Taxol, which was attributed to the PEG fictionalization. PTX uptake of tumor for SWCNT-based PTX delivery system is likely through an enhanced permeability and retention effect (EPR effects). Hepatic macrophage showed considerable uptake [91], but histopathological and biochemical studies found no obvious morphological changes, although the function of the liver requires examining for any potential side effects of SWCNT-PTX.

Wu et al. successfully constructed a novel MWCNT-based drug delivery system by tethering anticancer agent hydroxycamptothecin (HCPT) onto the surface of MWCNTs. By carboxyl enrichment via optimized oxidization treatment, CNTs were surface-functionalized, which was followed by amidation with a hydrophilic diaminotriethylene glycol, and subsequent conjugation of succinylated HCPT to hydroxyl derivatized MWCNTs was achieved via a cleavable ester linkage. In comparison with the clinical, presently used HCPT formulation, the MWCNT-HCPT complexes demonstrated superior antitumor activity and low toxicity both *in vitro *and *in vivo*. The prepared complexes had longer blood circulation time and higher tumor-specific drug accumulation as *in vivo *single photon emission computed tomography imaging and *ex vivo *g-scintillation counting analysis disclosed. These properties synergistically boost the antitumor efficacy of the conjugate [92]. Through *in vivo *observation of the killing effects on cancer cells, Bhirde et al. demonstrated superior efficacy of drug-SWCNT bioconjugates over nontargeted bioconjugates [93]. In order to specifically target squamous cancer, anticancer agent cisplatin and epidermal growth factor (EGF) were attached to SWCNTs. SWCNT-cisplatin without EGF was used as a nontargeted control. It was revealed that SWCNT-quantum dot-EGF bioconjugates internalized rapidly into the cancer cells as confirmed by imaging studies with head and neck squamous carcinoma cells (HNSCC) overexpressing EGF receptors (EGFR) using quantum dot (linked covalently) luminescence and confocal microscopy. Control cells showed limited uptake and cellular uptake can be blocked by siRNA knockdown of EGFR, revealing the importance of EGF in the system. Imaging with three color, two-photon intravital video showed that injected SWCNT-quantum dot-EGF was selectively taken up by HNSCC tumors causing rapid regression, but in those control animals, SWCNT-quantum dot was cleared from the tumor region in less than 20 min. HNSCC cells were also killed selectively by SWCNT-cisplatin-EGF, while control systems caused no effect on the proliferation of these cells [93].

Dendritic cells are of importance for the induction and regulation of immune responses. To carry siRNA into the antigen-presenting dendritic cells *in vivo*, cationic 1,6-diaminohexane functionalized CNTs were prepared. Splenic CD11c+ dendritic cells, CD11b+ cells and also Gr-1+CD11b+ cells comprising dendritic cells, macrophages, and other myeloid cells showed active take up for the complexes. The complexes silenced suppressor of cytokine signaling 1 (*SOCS1*) expression and retarded the growth of B16 tumor in mice [94]. It is well known that telomerase reverse transcriptase plays a critical role in tumor development and growth through the maintenance of telomere structure. The same group of authors coupled siRNA to SWCNTs via a CONH-(CH_2_)_6_-NH_3_^+ ^Cl^- ^spacer, demonstrating that siRNA delivered via SWCNT complexes silences the expression of telomerase reverse transcriptase and inhibits the proliferation and growth of tumor cells both *in vitro *and in mouse models. By 48 h, all of the cells, such as LLC, TC-1, and 1H8 cells, treated with telomerase reverse transcriptase SWCNT-siRNA showed an almost complete inhibition of proliferation in murine tumor models [95]. Unmodified siRNA with pristine SWCNTs have also been complexed noncovalently to deliver iRNA to cancer cells in a more recent study. The complex was prepared by simple sonication of pristine SWCNTs in a solution of siRNA that plays the dual role of cargo and dispersant for CNTs. It was envisioned that there was strong specific inhibition of cellular HIF-1 activity when siRNA complexes targeted to hypoxia-inducible factor 1 a (HIF-1a) were added in serum-containing culture media. The ability to response biologically to SWCNT-siRNA complexes have been observed in a wide variety of cancer cell types. Moreover, the activity of tumor HIF-1a was significantly inhibited by intratumoral administration of SWCNT-HIF-1a siRNA complexes in mice bearing MiaPaCa-2/HRE tumors, suggesting that such SWCNT-siRNA complexes promise considerably as therapeutic agents [96].

#### Drug delivery targeted to central nervous system

**Figure 4:**
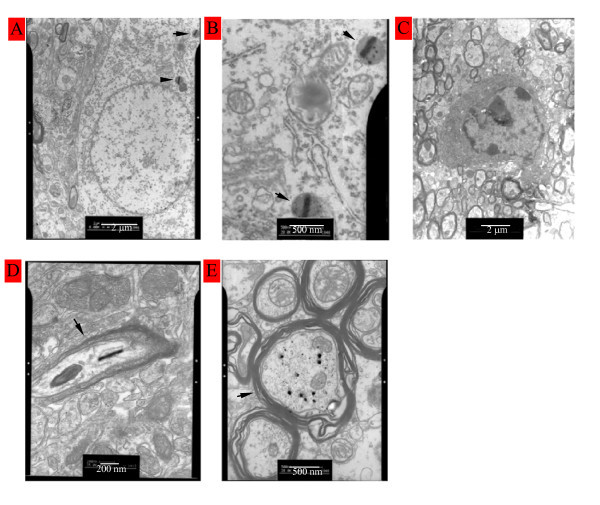
**SWCNTs enter the neurons in brain through axoplasmic transportation **[10]. (**A**) SWCNTs in the lysosomes of a neuron (arrows); (**B**) the magnification of the two lysosomes containing SWCNTs; (**C**) there are no SWCNTs in glial cells; (**D**) a bundle of SWCNTs parallel to the neurite in the section along the longitudinal axis of the neurite; (**E**) the SWCNTs are dot-like in the section vertical to the longitudinal axis of the neurite.

To deliver drugs to central nervous system is still a serious challenge in anticancer drug delivery system for the treatment of the tumors in the central nervous system because of the blood-brain barrier. Recently, our group devised a simple drug delivery system of acetylcholine for treatment of Alzheimer disease. Acetylcholine is natural neurotransmitter of the cholinergic nervous system and related with high-level nervous activities, such as learning, memory, and thinking. Because of the synthesis impairment, acetylcholine is decreased in the neurons in the Alzheimer disease brain, leading to the incapability of learning, memory, and thinking. Providing acetylcholine for the neurons would be able to prove the intellectual activities of the patients with Alzheimer disease. But there have been no way to deliver acetylcholine into brain because the acetylcholine is a compound with strong polarities, making it difficult to pass through the blood-brain barrier. In our newly prepared drug delivery system, the adsorption of acetylcholine on SWCNT was confirmed by Raman spectrum, although it was unknown whether acetylcholine was adsorbed on the surfaces or in the tubes of SWCNT. This system successfully delivered acetylcholine into the neurons in the brain through the axoplasma transformation of neurites (Figure 4) and significantly improved the learning and memory capabilities of the model animals with Alzheimer diseases [10]. This delivery system provided the first instance for nanocarriers to deliver drugs into neurons in the central nervous system, opening the door for the drug delivery system for the treatment of the tumors in central nervous system.

As regards the *in vivo *anticancer drug delivery system, it should be mentioned that the results of CNT-based drug delivery has been compared with some commercially available formulations. The results demonstrated several advantages of CNTs over other nanomaterial-based drug delivery systems. For example, Liu et al. compared the efficacy and the toxicity of doxorubicin-loaded CNTs with that of doxorubicin-loaded nanoliposomes [97]. However, a decisive conclusion has not been reached about whether CNTs are the best carriers in anticancer drug delivery systems because there are many other anticancer drug delivery systems based on other nanomaterials, such as cationic polymers [98], fibrinogens [99], and PVP/PVAL nanoparticles [100], that have also shown great promises as drug carriers for cancer treatments. More comparison works are required to make sure of the position of CNTs as drug carriers in anticancer therapies. In addition, the reproducibility of pharmacokinetic data is severely challenged by the facts that the volume, diameter, and length of CNTs are not well controlled in the studies, nor is there any reliable data on their size distribution. The commercialization of drug delivery systems based on CNTs will be impeded by these problems. To the best of our knowledge, no such system has gone into clinical trial and many difficulties are yet to be overcome before successful application of CNT-based drug delivery systems in practical therapy comes into fruition.

### The biosafety of SWCNT used as drug carriers

Although there are exciting prospects for the application of CNTs as drug carriers in medicine, one of the key obstacles in the way for the use of SWCNT as drug carriers is their biosafety. This problem has been quite controversial. There are many studies that believe SWCNT is safe, while many literatures have reported the damage effects on cells *in vitro *and on tissues *in vivo*. Fortunately, excitement encompassing the attractive physicochemical properties of CNTs has been tempered by issues concerning their potential health hazards, despite some toxic effects of CNTs have been observed, which give rise to concern about the biosafety of CNTs. The observed toxicity has been largely attributed to the structural analogy between CNTs and asbestos fibers in the high aspect ratio, large surface area, induction of inflammation [101-103], fibrosis [104-106], and malignant mesothelioma [107,108] on inhalation, and, more importantly, their biopersistence [109-111]. A recent *in vitro *study has revealed that there are close relations between the biodurability of CNTs and the chemistry of CNT surface functionalization. However, it was revealed by recent investigations that the toxicity of SWCNTs *in vivo *is resulted from the aggregation rather than the large aspect ratio of individual CNTs [112-114]. The experiment demonstrated that granuloma-like structures with mild fibrosis observed in mice treated with aggregated SWCNTs were completely absent in mice treated with nanoscale dispersed SWCNTs, even after 30 days of intratracheal administration. Histopathological sections from the lung of the mice treated with nanoscale dispersed SWCNTs revealed uptake of the SWCNTs by macrophages and demonstrated the gradual clearance over time, corroborating the biosafety and biocompatibility of CNTs in the form of nanoscale dispersions in *in vivo *applications. Further attempt has been tried to evaluate the toxicity of SWCNTs in Swiss mice as a function of dose, length, and surface chemistry. Some researchers have observed that oral administration of CNTs, even at very high doses (1,000 mg/kg bodyweight), induces neither death, growth, nor any behavioral dilemma. When intraperitoneally administered, SWCNTs were found to coalesce inside the body, forming fiber-like structures. It was further observed that smaller aggregates almost induced no granuloma. Rafeeqi and Kaul explored the interactions between multi-walled carbon nanotubes and cell culture medium by spectroscopy, and the results supported biocompatibility of these nanotubes [115]. From these literatures, both positive and negative biological effects of SWCNTs can be seen, which has been attributed to the methods used in the experiments. For example, Mittal et al. found that clear interference of CNTs with conventional *in vitro *cytotoxicity assays (MTT, NRU, and LDH) was found, which was confirmed by cellular system, but morphological changes, and flow cytometry showed the characteristics of cytotoxicity [116]. Brown et al. reported that the cytotoxicity of CNTs is related to their lengths [117].

**Figure 5:**
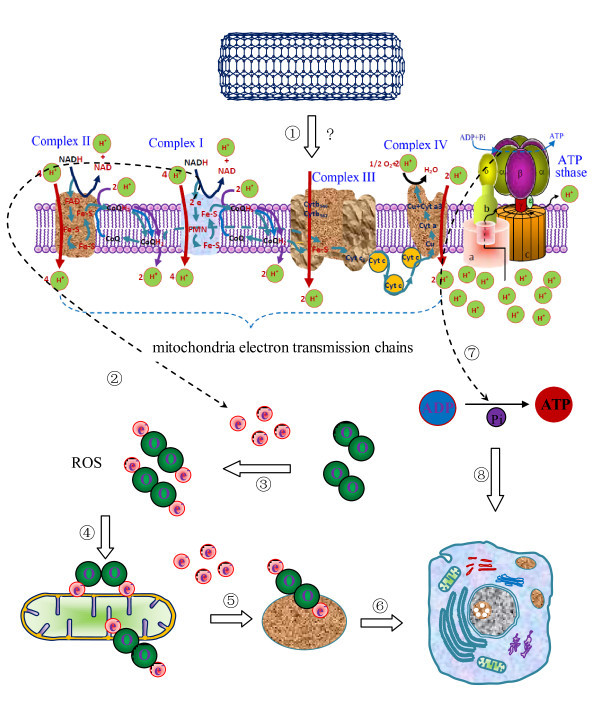
**The schematic illustration of the mechanisms of SWCNT to induce cell damage**. Based on the studies of Yang et al. [10]. SWCNTs interact with the mitochondria electron transmission chains (ETC) (by binding to ETC?) after they enter into mitochondria (1); The interaction of SWCNTs with ETC blocks the transmission of electrons, which results in the increase of the leaking of free electrons from ETC (2); The leaked free electrons form free radicals H_2_O_2 _or reactive oxygen species (ROS) (3); The free radicals or ROS attack the membrane system of mitochondria through peroxidation (4); Then the free radicals or ROS diffuse through the damaged mitochondrial membrane to lysosomes to destroy the membrane of the lysosomes (5); The injured lysosomes release digestive enzymes, leading to the damage or death of the whole cells. On the other hand, the blocking of ETC makes mitochondria incapable of producing ATP (7), which results in the depletion of energy for the living activities of the cells, also leading to the damage or death of the whole cells (8).

More recently, we carefully and extensively investigated the biosafety problem of SWCNTs [10]. The results demonstrated that SWCNT are considerably safe with a safe range of 12 as revealed by the ratio of ED99/LD99, where the ED99 is the doses effective for half of the model animals with Alzheimer disease. It was also demonstrated that the pharmacological and toxicological effects of SWCNT are mediated separately by lysosomes and mitochondria. *In vitro*, SWCNT induced the increase of reactive oxygen species (ROS) in lysosomes while had no influences on the ROS level in mitochondria. *In vivo*, SWCNT exclusively distributed in lysosomes when the doses was under 300 mg/kg, but there was no ultrastructural damage as revealed by transmission electron microscope. SWCNT began to enter mitochondria when the doses got over 400 mg/kg. Once SWCNT entered, the pathological ultrastructural changes developed in mitochondria. The mitochondria dilated, the crestae decreased or disappeared, and even the whole mitochondria became a vacuole. Once the mitochondria damaged developed, the lysosomes also appeared as damaged. The membrane of lysosomes destroyed, the contents of lysosomes decreased or excreted, and even the whole lysosomes became a huge vacuole. All of these results indicated that mitochondria are the original organelles for SWCNT damage and the lysosomal damage is secondary to mitochondrial damage, namely, mitochondria are the toxicological target organelles and lysosomes are the pharmacological target organelles of SWCNT. Further experiments demonstrated that the damage of SWCNT on mitochondria was resulted by its influences on the mitochondrial membrane potentials (MMP). The decrease in MMP means there is leakage of free electrons, which results in the increase of the ROS, further leading to the ultrastructural damage of the cells including lysosomes. The mechanism of SWCNT damage can be summarized in Figure 5.

The progress in several aspects of the safety studies on SWCNT is of great importance for its practical use as drug carriers for cancer treatment. Firstly, the modification or functionalization can significantly decrease the toxicity of CNTs, making it possible to select safe CNT derivatives as drug carriers. Secondly, it has been found that CNTs can be metabolized in liver and eliminated through kidneys and hap-bile systems, making less concern about the persistence residence of them in bodies. Thirdly, it has been illustrated that the pharmacological and the toxicological effects of CNTs are mediated by different target organelles and the distribution of CNTs in organelles can be regulated by some chemicals, making it possible to use the advantage and decrease or avoid the disadvantage of them. Fourthly, the dose differences exist between the pharmacological and toxicological effects of CNTs, which means that the toxicological effects may be avoid by controlling the doses. Through these progresses, it can be predicted that the safety problems will be solved in not too long a time.

### The future of CNTs used as drug carriers for cancer treatments

Summarizing the above described progress in the studies on the application of CNTs as drug carriers, it may be seen that the chemistry on the modification of CNTs now has considerably grown up. The previous studies have provided us various chemical methods to solve some fundamental problems in the use of CNTs as drug carriers such as their water solubility and target properties. Many reported results, including those obtained from *in vitro *and *in vivo *experiments, have demonstrated that CNTs can increase the treatment effects while decrease the side and toxic effects of the drugs loaded on them, indicating a considerably bright future for them to be used as drug carriers. However, there is a long way to go for CNTs to get into practical use. Particularly, the pharmacological and toxicological profiles must be made completely clear and the advantages and the disadvantages of CNTs must be carefully weighed before they are used as drug carriers in human body.

**Figure 6:**
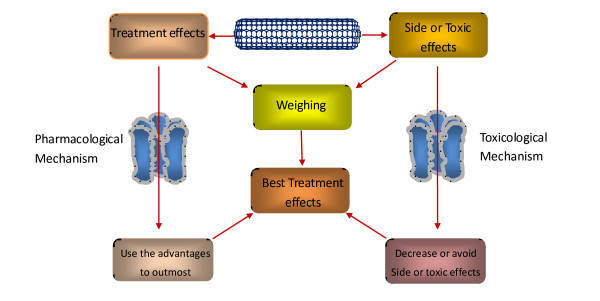
**The schematic illustration of the strategies**. For the studies on the best use of CNTs as drug carriers. The best treatment effects are in the center of the strategies that are the ultimate purpose of our studies, which can be achieved by the studies of three effects: the weighing between treatment effects and the side or toxic effects, the pharmacological mechanism that makes it possible to use the advantages to outmost and the toxicological mechanism that makes people capable of decreasing or avoiding the side or toxic effects.

On the bases of matured chemical modification, the remaining key to the successful practical use of CNTs as drug carriers is to make clear of the mechanisms for their pharmacological and toxicological effects. The understanding of the pharmacological mechanisms makes it possible to take the advantage of CNTs to the outmost and to avoid or limit the disadvantages to possibly low degree (Figure 6). The weighing of the advantage and disadvantage in the treatment of a special disease is also very important because CNT-based drug delivery system also has its indication and contraindication just like any other drugs.

After all, the studies on the application of CNTs as drug carriers for the treatment of cancers have achieved important progress. Some key obstacles in the way to practical use have been overcome. Although there is still a long way to go for the practical use, it may be predicted that, on one day in the future, CNTs will become an important class of drug carriers for cancer treatment.

## Competing interests

The authors declare that they have no competing interests.

## Authors' contributions

ZWX drafted the manuscript. ZZZ and ZYG go over and corrected the manuscript. All authors read and approved the final manuscript.
